# Optimizing HIV pre-exposure prophylaxis implementation among men who have sex with men in a large urban centre: a dynamic modelling study

**DOI:** 10.7448/IAS.19.1.20791

**Published:** 2016-09-23

**Authors:** Derek R MacFadden, Darrell H Tan, Sharmistha Mishra

**Affiliations:** 1 Division of Infectious Diseases, Department of Medicine, University of Toronto, Toronto, Ontario, Canada; 2 Division of Infectious Diseases, Department of Medicine, St. Michael's Hospital, Li Ka Shing Knowledge Institute, Toronto, Ontario, Canada; 3 Department of Infectious Disease Epidemiology, Imperial College, London, United Kingdom

**Keywords:** HIV, PrEP, cost-effectiveness, dynamic model, prevention, prophylaxis

## Abstract

**Introduction:**

Once-daily tenofovir/emtricitabine-based pre-exposure prophylaxis (PrEP) can reduce HIV acquisition in men who have sex with men (MSM), by 44% in the iPrEx trial, and reaching up to 99% with high adherence. We examined the potential population-level impact and cost-effectiveness of different PrEP implementation strategies.

**Methods:**

We developed a dynamic, stochastic compartmental model of HIV transmission among the estimated 57,400 MSM in Toronto, Canada. Parameterization was performed using local epidemiologic data. Strategies examined included (1) uniform PrEP delivery versus targeting the highest risk decile of MSM (with varying coverage proportions); (2) increasing PrEP efficacy as a surrogate of adherence (44% to 99%); and (3) varying HIV test frequency (once monthly to once yearly). Outcomes included HIV infections averted and the incremental cost ($CAD) per incremental quality-adjusted-life-year (QALY) gained over 20 years.

**Results:**

Use of PrEP among all HIV-uninfected MSM at 25, 50, 75 and 100% coverage prevented 1970, 3427, 4317, and 4581 infections, respectively, with cost/QALY increasing from $500,000 to $800,000 CAD. Targeted PrEP for the highest risk MSM at 25, 50, 75 and 100% coverage prevented 1166, 2154, 2816, and 3012 infections, respectively, with cost/QALY ranging from $35,000 to $70,000 CAD. Maximizing PrEP efficacy, in a scenario of 25% coverage of high-risk MSM with PrEP, prevented 1540 infections with a cost/QALY of $15,000 CAD. HIV testing alone (Q3 months) averted 898 of infections with a cost savings of $4,000 CAD per QALY.

**Conclusions:**

The optimal implementation strategy for PrEP over the next 20 years at this urban centre is to target high-risk MSM and to maximize efficacy by supporting PrEP adherence. A large health benefit of PrEP implementation could come from engaging undiagnosed HIV-infected individuals into care.

## Introduction

HIV incidence among men who have sex with men (MSM) in high-income countries remains high and concentrated in large, urban settings [1–6]. In Canada, the incidence of HIV infection among MSM ranges from 0.62 per 100 person-years to 1.14 per 100 person-years, similar to the ranges in other developed countries [2–6]. In 2014, 837 new HIV infections were diagnosed in the province of Ontario, with half occurring in Canada's largest city, Toronto [5,6]. Of the estimated 57,400 MSM living in Toronto, nearly 20% have HIV [1]. Despite the scale-up of a combination anti-retroviral therapy (ART) and sustained investments in behavioural prevention programmes [7,8], the rates of newly diagnosed HIV infections and HIV-attributable deaths in Toronto MSM have not markedly declined over the last 10 years, and remain a major public health concern [1]. Daily use of tenofovir/emtricitabine (Truvada ^®^) by HIV-uninfected individuals as pre-exposure prophylaxis (PrEP) has been shown in randomized trials to reduce HIV acquisition in high-risk groups [9–11]. The iPrEx study, a placebo-controlled trial in high-risk MSM, found that those randomized to tenofovir/emtricitabine were 44% less likely to acquire HIV compared to placebo over a median follow-up of 1.2 years [9], and pharmacokinetic analyses suggest efficacy of up to 99% if adherence is high [12]. More recent data from the iPrEx open-label extension corroborate this estimate, with dosing of four to seven times per week associated with virtually 100% (95% CI=86,100%) efficacy [13]. To inform broader PrEP implementation, additional demonstration projects are underway across North America, including the US PrEP community-based demonstration project out of New York City, an NIH-funded community-based project out of San Francisco [14], and PREPARATORY-5 (NCT02149888), a demonstration project in Toronto evaluating PrEP acceptability, effectiveness and sexually transmitted infection rates. To contextualize the findings of these regional demonstration projects and prepare for wide-scale PrEP delivery, we need to understand how to maximize its population-level impact on HIV transmission in large, urban centres, given the added cost to the healthcare system.

Dynamic mathematical models of the population impact of PrEP on HIV spread among MSM have been developed on both national and sub-national scales in high- [15–19] and low-income regions [20]. Five modelling studies of high-income countries suggest that PrEP is most cost-effective when restricted to the highest risk subgroups [15–19]. Not surprisingly, these studies also showed that uptake and adherence would have large effects on predicted HIV outcomes. However, key implementation questions remain unanswered. For instance, although clinical guidelines recommend that HIV testing be done every three months in individuals using PrEP on the basis of the protocols used in clinical trials, the optimal HIV screening frequency remains unclear. It is also unclear what proportion of the total benefit of PrEP programmes accrues from the ability of PrEP-related HIV testing to diagnose infections earlier, versus from the use of PrEP itself. Furthermore, most models of PrEP population-effectiveness and/or cost-effectiveness in high-income settings examined national-level epidemics by drawing on national-level sexual behaviour and HIV surveillance data [15,17–19]. However, a national perspective belies the heterogeneity in HIV epidemics between locales, such as states, provinces or major cities [4]. Furthermore, HIV prevention programmes are often funded and administered at a regional level [6,21]. There is often marked heterogeneity of prevalence and rates of HIV infection/diagnosis across regions within countries, and comparing metropolitan and non-metropolitan regions [22,23]. Regional models of PrEP may thus offer a more relevant assessment of implementation costs and outcomes for the purpose of guiding local interventions, and may be more generalizable to regions with similar epidemic characteristics. To date, there has been no model-based evaluation of PrEP implementation in a Canadian city.

To address these gaps, we developed a mathematical model of HIV spread in Toronto MSM using the best available, regional epidemiologic data. Our aim was to evaluate the impact of different strategies of PrEP delivery in Toronto MSM on the following outcomes: reduction in the total number of diagnosed and undiagnosed HIV infections, total number of HIV-related deaths averted, incremental costs and cost per quality-adjusted life year (QALY) gained, over a 20-year period. Strategies examined included targeting PrEP at the highest risk MSM (with varying proportions of coverage) and varying the frequency of HIV screening in those on PrEP. We further assessed the sensitivity of outcomes to varying rates of adherence.

## Methods

### Model design

We developed a dynamic, deterministic-stochastic hybrid compartmental model of HIV spread among MSM in Toronto, Canada. The model was represented by a combined deterministic-stochastic model [24] of state-transitions based on the probability of moving from one compartment to another. The transitions were stochastic for compartment population sizes fewer than 5000 individuals, and were deterministic for compartments exceeding 5000 individuals. The motivation for using a stochastic approach was to better accommodate relatively small population sizes within compartments that would be encountered in a regional model. A deterministic function was used to accommodate the few compartments that would have a consistently higher population, allowing improved computational efficiency. For this analysis, we chose to present mean values, corresponding to repeated realizations of the model.

**Figure 1:**
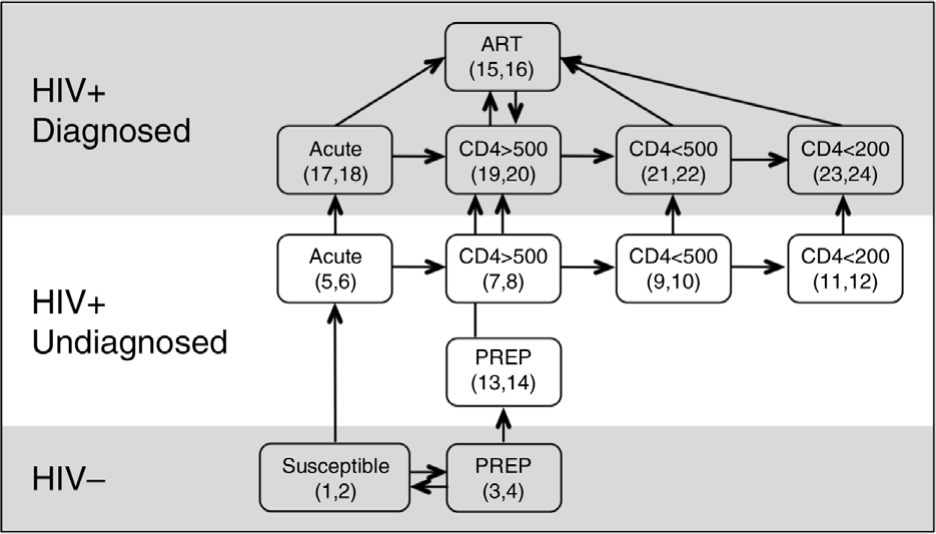
A schematic of the model compartments and intercompartmental flow, organized by serostatus (known/unknown). *Not denoted are the entry of individuals into the population as susceptible, and the exit of individuals out of the population. **Compartment numbers are listed in brackets for simplicity of reference with model equations. Odd numbers refer to high sexual activity/risk compartments, and even numbers refer to low sexual activity/risk compartment.

Figure 1 shows the schematic of the model structure, while Table 1 shows the parameter values. Model compartments reflected the natural history (or “states”) of untreated and treated HIV, and populations were further stratified by known/unknown HIV serostatus and sexual behaviour (high/low risk). The natural history of untreated HIV was divided into four progressive stages reflecting data from untreated HIV-infected cohorts [25]. Each stage was associated with a different probability of infectiousness and HIV-attributable mortality (Table 1), and defined by time to specific CD4 cell counts [25]: (1) acute seroconversion; (2) CD4>500 cells/mm^3^ as early disease; (3) CD4 200 to 500 representing late HIV and (4) CD4<200 representing AIDS. The model represented an open population of MSM. HIV-uninfected men entered the model at a rate maintaining 1% population growth. HIV-uninfected and HIV-infected MSM left the modelled population at compartment-specific rates, with mortality dependent upon the stage of infection.

**Table 1:** Model parameter estimates and ranges^a^

Variable	Value used
Biologic	
Model exit rate (δ) [28]	0.0043
Model death rate due to AIDS (δ_*a*_, *i*=11,12,23,24) [15]	0.21 (0.2 to 0.4)
Population growth rate	0.01 (0 to 0.05)
HIV progression rate per year (*η*_*i*_) [25]	
Acute to CD4>500 (*i*=5,6,17,18)	4
CD4>500 to CD4<500 (*i*=7,8,19,20)	0.844
CD4<500 to CD4<200 (*i*=9,10,21,22)	0.167
Probability of HIV transmission(τ_*j*_) [15,29–31]	
Acute (transmissions per partnership per year) (*j*=5,6,17,18)	0.21
CD4>500 (transmissions per partnership per year) (*j*=7,8,19,20)	0.039
CD4<500 (transmissions per partnership per year) (*j*=9,10,21,22)	0.039
CD4<200 (transmissions per partnership per year) (*j*=11,12,23,24)	0.16
Behavioural	
Annual rate HIV testing, non-HIV-infected MSM, not on PrEP (per year) (ζ) [1]^b^	0.22
Combined condom use and efficacy term (*ω*_*j*_, *j*=1 to 16) [1,40]	0.78 (0.6–0.95)
Increase in combined condom use and efficacy after diagnosis (*ω*_*j*_, *j*=17 to 24) [27]	1.47
Proportion of MSM defined as low risk	0.9
Annual number of partnerships (among low risk)(π_*i*_, *i*=even numbers)	5
Proportion of MSM defined as high risk	0.1
Annual number of partnerships (among high risk) (π_*i*_, *i*=odd numbers)	36
Cost	
Annual PrEP drug cost per patient (CAD$) [15,36,38]	10,012
PrEP clinic cost per visit (CAD$) [37]	
Initial visit	305
Subsequent visits	100
Annual clinical care cost per patient (CAD$) [36,38]	
HIV+ (no ART)	8854
AIDS (no ART)	13,814
HIV+ (on ART)	15,264
HIV diagnostic testing costs per test (CAD$) [37]	
Negative ELISA	19
Positive ELISA+Western	82
Quality of life [15,38]	
On PrEP	1
Unidentified HIV+ (CD4>200)	0.91
Identified HIV+ (CD4>200)	0.89
Unidentified AIDS	0.73
Identified AIDS	0.73
On ART	0.83
Treatment	
PrEP efficacy (%) (ɛ) [9]	44 (44 to 99)
ART cessation rate per year (ϱ)	0.14 (0–0.4)
PrEP cessation rate per year (ϖ)	0
PrEP initiation rate per year (ϕ)	0.25
On treatment rate per year (α_*i*_) [40]	
Acute (*i*=5)	0.31 (0–0.5)
CD4>500 (*i*=7)	0.31 (0–0.5)
CD4<500 (*i*=9)	0.78 (0.5–1)
CD4<200 (*i*=11)	0.78 (0.5–1)

a Unless otherwise specified, all units are per year. Subscripts (*i* and *j*) denote compartment of reference (See Figure 1)

b Annual testing rate derived based on number of HIV tests performed annually in MSM within the City of Toronto and the size of the susceptible MSM population.

The sexual behaviour of high-risk MSM was drawn from the annual number of sexual partners taken from regional empiric data (using the highest decile of the reported number of sexual partners in the last year). These data were collected from MSM undergoing anonymous HIV testing at a downtown Toronto sexually transmitted infection clinic in Spring 2013 and suggested that the highest decile of MSM had a mean of 36 partnerships in the last year [26]. The remainder had a mean of five partnerships in the last year. For this aforementioned study, research ethics board approval was obtained from the University of Toronto, protocol #30129. Consent for the use of these data was obtained at the time of entry into the study. Data analysis performed for this study was done using anonymous and deidentified data.

HIV serostatus was based on an individual's awareness of his diagnosis of HIV. We assumed that HIV infectivity decreased after a diagnosis of HIV infection, based on behaviour change observed in population-based studies [27]. The reduction in infectivity was operationalized as a reduced probability of transmission between HIV-infected and uninfected individuals, reflecting either increased use of condoms or seropositioning favouring lower transmission, but not a change in the number of partners.

HIV transmission between compartments was a function of the following variables (Table 1): (1) the number of sexual partners per individual per annum; (2) condom use, which was combined with condom efficacy; (3) HIV transmission probability based on HIV stage, awareness of HIV serostatus and HIV treatment status and (4) PrEP use, which decreased HIV susceptibility (per sex act) but did not impact on the number of partners.

Individuals who were diagnosed with HIV could initiate ART at a rate that varied depending upon CD4 stage, as described below. Similarly, individuals who were on ART could discontinue treatment and return to the diagnosed, infected, but untreated population with CD4>500. In the intervention scenarios, PrEP could be initiated among HIV-uninfected individuals. If taking PrEP, the likelihood of acquiring HIV infection per act was reduced by 44% (unless otherwise evaluated). If individuals became infected with HIV while on PrEP, they were infectious while undiagnosed (with no effect of PrEP on HIV infectivity). Once diagnosed, they moved into the diagnosed HIV category. We did not incorporate the potential impact of PrEP use on emergence of antiretroviral drug resistance.

### Epidemiological and behavioural data for model parameterization

Toronto-specific epidemiologic data was used wherever possible for parameters and initial conditions (Table 1, Supplementary Tables 1 and 2). Baseline (non-HIV) mortality rates were based on average North American life expectancies [28], and adjusted based on expected increased mortality in late-stage (CD4<200) HIV infection [15]. The probability of HIV transmission, as a function of the stage of CD4 count (transmissions per partnership per year) was drawn from the literature [15,29–31]. The annual HIV testing rate among MSM in Toronto was estimated by dividing the mean annual number of HIV tests performed in Toronto MSM by the estimated population of undiagnosed MSM [1]. We used an annual 1% population growth rate of susceptible MSM, in keeping with Canadian population growth rates [32]. Individuals on ART were assumed to be fully adherent and non-infectious [33]. Estimated rates of condom use and efficacy, used for calibration, were based on published values and local data [1,34,35].

**Table 2:** Estimated population-level outcomes at 20 years after universal versus focused introduction of PrEP among MSM

Outcome	Base case	% of all HIV-uninfected (high- and low-risk MSM) on PrEP				% of high-risk MSM on PrEP			
	
		25%	50%	75%	100%	25%	50%	75%	100%
Cumulative HIV outcomes									
New HIV diagnoses	7,181	6,119	5,318	4,838	4,730	6,480	5,906	5,537	5,444
New (incident) HIV infections	8,378	6,409	4,951	4,062	3,797	7,212	6,224	5,563	5,366
HIV infections prevented	–	1,970	3,427	4,317	4,581	1,166	2,154	2,816	3,012
Mortality (HIV-related)									
Total	1,567	1,457	1,385	1,344	1,330	1,497	1,450	1,430	1,427
Prevented	–	110	182	222	236	70	117	137	140
Costs (CAD)									
Untreated HIV total cost	2.38E+08	2.29E+08	2.22E+08	2.18E+08	2.18E+08	2.36E+08	2.27E+08	2.23E+08	2.22E+08
HIV treatment total cost	1.74E+09	1.71E+09	1.68E+09	1.67E+09	1.67E+09	1.72E+09	1.70E+09	1.69E+09	1.69E+09
PrEP total cost	–	1.36E+09	2.65E+09	3.80E+09	4.37E+09	7.98E+07	1.62E+08	2.39E+08	2.69E+08
Susceptible testing cost	2.20E+06	1.70E+06	1.20E+06	7.48E+05	5.22E+05	2.19E+06	2.18E+06	2.16E+06	2.18E+06
Total cost	1.98E+09	3.31E+09	4.57E+09	5.72E+09	6.23E+09	2.03E+09	2.09E+09	2.16E+09	2.18E+09
Incremental QALYs (years)	–	2,673	4,413	5,363	5,430	1,417	2,321	3,080	2,951
Incremental cost-effectiveness (CAD)									
Cost/diagnosed infection averted	–	1,246,500	1,390,681	1,593,890	1,756,263	70,667	85,203	105,055	115,888
Cost/total infection averted	–	671,857	755,918	865,041	939,657	42,508	50,431	61,359	66,809
Cost/HIV-associated death averted	–	12,059,137	14,249,319	16,784,874	18,218,284	711,931	931,723	1,265,545	1,440,334
Cost/QALY gained	–	495,175	587,050	696,297	792,763	34,999	46,818	56,084	68,203

### Cost and quality of life data

Cost-effectiveness analysis was conducted from the health-systems perspective and included costs associated with inpatient and outpatient clinical care and diagnostics. PrEP, ART and HIV/AIDS related care costs were drawn from previous studies based on Canadian estimates, and included physician-visit and nursing costs, acute hospitalization costs, diagnostic testing and prescription costs [36,37]. Specific diagnostic testing costs in susceptible individuals were determined based on provincial (Ontario) cost listings for HIV serology as of July 2013 [37]. Estimates of stage-specific QALYs lost/gained among persons with HIV/AIDS and those on ART were determined from the literature [38]. We assumed that PrEP did not affect quality of life. In evaluating cost-effectiveness, we determined the total health care related costs, as well as costs associated with PrEP implementation, HIV treatment related costs, untreated HIV-associated costs, HIV testing costs among the susceptible population and QALYs. QALYs were calculated at each time step of the model. Costs and QALYs were discounted at a rate of 3% per annum.

### Model calibration

We calibrated the model at an equilibrium state against the following historical estimates (10-year period in Toronto, 2001 to 2011) using acceptance-rejection sampling: (1) a stable annual HIV diagnosis rate (330 to 400 cases per annum) and (2) a stable annual HIV-attributable mortality (58 to 72 cases annually) [1,39]. Parameter values were selected using Monte-Carlo sampling. The calibrated parameters are listed in Table 1, and included those about which we were most uncertain and those expected to influence study outcomes the most: ART initiation and cessation rates, reduction in risky sexual behaviour after HIV diagnosis, AIDS mortality, condom use and efficacy. We cross-validated the model-generated HIV prevalence ratio (high-risk versus low-risk population), against local data on HIV prevalence among the 10% of MSM with the highest risk of HIV (using syphilis co-infection as a risk factor) compared to the lowest-risk 90%, which was a ratio of 3 [35].

### Analyses

We used the accepted parameter sets to simulate the baseline scenario (without PrEP), and different strategies of PrEP delivery. PrEP was introduced at an HIV-equilibrium state and outcomes were measured at 20 years. Because we were interested in the mean outcome per parameter set (*i.e*., the average of a large number of stochastic realizations), we performed 150 stochastic realizations per parameter set to ensure the mean incidence of diagnosed and undiagnosed HIV infections varied less than 1% with each additional simulation.

Outcomes included the impact of daily PrEP on cumulative number of newly diagnosed and undiagnosed HIV infections, the cumulative number of HIV-associated deaths, annual costs and cost per QALY. Newly diagnosed infections included those individuals who were already infected at the start of the simulation, and then were subsequently diagnosed. We compared the following PrEP implementation strategies: (1) targeting all MSM (high- and low-risk sexual activity) versus only high-risk activity MSM alone, with varying proportions of coverage; (2) varying the frequency of HIV testing in those taking PrEP including the scenario in which screening is performed without PrEP use and (3) increasing PrEP efficacy (as a surrogate for adherence) based on pharmacokinetic analyses from the iPrEx trial, from 44% risk reduction (corresponding to fewer than two doses per week), to 76% (two doses per week), 96% (four doses per week), and 99% risk reduction (seven doses per week) [12]. We then conducted a sensitivity analysis to explore the influence of changing coverage in an ideal adherence scenario (99% efficacy).

## Results

In the absence of PrEP, the model estimated 8378 new HIV infections and 1567 HIV-attributable deaths over 20 years. Of the new HIV infections, 30% were acquired by high-risk MSM. The prevalence ratio of high to low-risk was 3, in keeping with the increased risk of infection in the high-risk population [35].

### Risk targeting

Over 20 years, PrEP use in 25, 50, 75 and 100% of MSM prevented 1970, 3427, 4317, and 4581 new HIV infections, and 110, 182, 222, and 236 HIV-associated deaths. The estimated cost of implementing PrEP increased as more men used PrEP, from $1.36 billion CAD with 25% PrEP coverage to $4.37 billion CAD with 100% PrEP coverage. Total QALYs gained varied from 2673 to 5430, and cost per QALY gained varied from $495,175 to $792,763 CAD with 25% to 100% PrEP coverage, respectively (Table 2).

**Figure 2:**
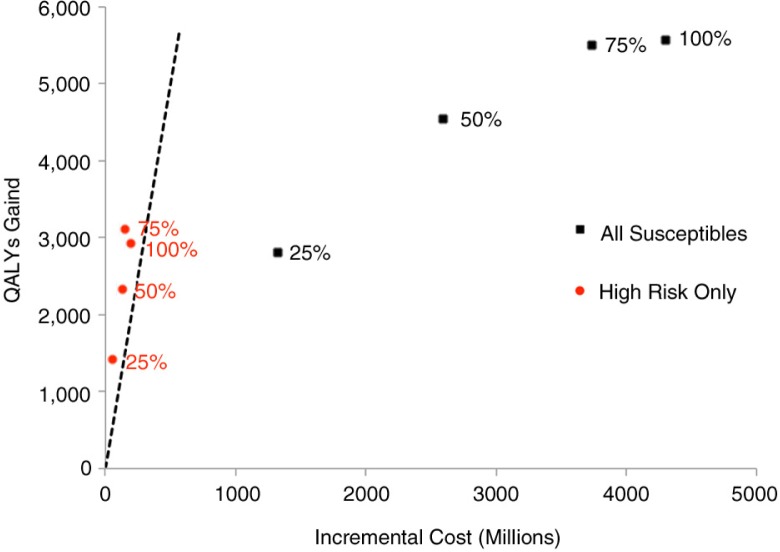
Cost-effectiveness of targeted PrEP implementation in varying proportions of high-risk MSM and all susceptible MSM. *All scenarios are compared to baseline (no PrEP use). **Dashed line represents upper threshold of cost-effectiveness (cost/QALY gained) in Canada ($100,000 CAD), where points to the left of the line are “cost-effective” [41].

In contrast, restricting PrEP to the highest risk decile of HIV-uninfected MSM at 25, 50, 75 and 100% coverage was estimated to prevent 1166, 2154, 2816, and 3012 new HIV infections, and 70, 117, 137, and 140 HIV-associated deaths over 20 years. The cost of targeting PrEP at the highest risk MSM increased from $80 million CAD with 25% PrEP coverage to $269 million CAD with 100% PrEP coverage. Total QALYs gained varied from 1417 to 2951, and cost per QALY gained varied from $34,999 to $68,203 CAD with 25% to 100% PrEP coverage respectively (Table 2). Incremental cost-effectiveness (cost per QALY gained) was greatest when PrEP use was restricted to the high-risk MSM (Figure 2).

Increasing PrEP coverage in high-risk MSM (25%, 50%, 75%, 100%) under the assumption of 99% efficacy (and ideal adherence scenario) prevented more infections (1540, 2881, 3827, 4095), prevented more HIV-associated deaths (87, 152, 182, 196), led to more QALYs gained (1852, 3286, 4033, 4108) and produced improved cost per QALY ($15,275, $24,099, $35,682, $44,427 CAD).

### Clinical implementation parameters

To evaluate the impact of HIV testing frequency and PrEP efficacy among PrEP users, we assumed a baseline scenario in which 25% of high-risk MSM used PrEP. When keeping efficacy fixed at 44%, changing the frequency of HIV testing in those on PrEP had minimal influence on the number of HIV infections prevented over 20 years (1209 to 1113 HIV infections prevented with Q1 month versus Q12 month testing, respectively). Similarly, costs and cost-effectiveness did not significantly change with variation in testing frequency (Table 3). However, when keeping test frequency fixed at Q3 months, increasing PrEP efficacy from 44% to 99% resulted in a marked increase in infections prevented, from 1166 to 1540. Cost-effectiveness improved with cost per QALY gained, decreasing from $34,999 to $15,275 CAD (Table 3). Finally, in the scenario of 25% coverage of high-risk HIV-uninfected MSM, we considered the option of HIV screening only and no PrEP administration. This resulted 898 infections prevented, 1164 QALYs gained and a cost savings per QALY gained of $4,332 CAD.

**Table 3:** Estimated population-level outcomes at 20 years after introduction of PrEP in 25% of high-risk HIV-uninfected MSM, with variable frequency of HIV testing in those on PrEP, with variable PrEP efficacy, and HIV testing only

Outcome	Base case	Testing frequency^a^						PrEP efficacy			
	
		Q1 month	Q2 months	Q4 months	Q6 months	Q12 months	Testing only^b^	44%	76%	96%	99%
Cumulative HIV outcomes											
New HIV diagnoses	7,181	6,453	6,457	6,479	6,496	6,504	6,737	6,480	6,284	6,121	6,114
New (incident) HIV infections	8,378	7,170	7,173	7,209	7,239	7,266	7,480	7,212	7,022	6,824	6,839
HIV infections prevented	–	1,209	1,206	1,169	1,140	1,113	898	1,166	1,357	1,555	1,540
Mortality (HIV-related)											
Total	1,567	1,494	1,503	1,500	1,508	1,503	1,519	1,497	1,493	1,483	1,480
Prevented	–	73	64	66	59	64	47	70	74	84	87
Costs (CAD)											
Untreated HIV total cost	2.38E+08	2.31E+08	2.31E+08	2.31E+08	2.31E+08	2.31E+08	2.35E+08	2.36E+08	2.28E+08	2.26E+08	2.26E+08
HIV treatment total cost	1.74E+09	1.72E+09	1.72E+09	1.72E+09	1.72E+09	1.72E+09	1.73E+09	1.72E+09	1.71E+09	1.70E+09	1.70E+09
PrEP total cost	–	7.99E+07	7.99E+07	7.98E+07	7.99E+07	8.01E+07	–	7.98E+07	8.22E+07	8.52E+07	8.56E+07
Susceptible testing cost	2.20E+06	2.19E+06	2.19E+06	2.19E+06	2.19E+06	2.19E+06	2.19E+06	2.19E+06	2.19E+06	2.20E+06	2.20E+06
Total cost	1.98E+09	2.03E+09	2.03E+09	2.03E+09	2.03E+09	2.03E+09	2.05E+09	2.03E+09	2.02E+09	2.01E+09	2.01E+09
Incremental QALYs (years)	–	1,431	1,404	1,326	1,485	1,389	1,164	1,417	1,608	1,805	1,852
Incremental cost-effectiveness (CAD)											
Cost/diagnosed infection averted	–	69,066	66,246	69,396	72,415	71,479	−11,359	70,667	40,803	27,051	26,523
Cost/overall infection averted	–	41,605	39,797	41,656	43,500	43,520	−5,613	42,508	26,984	18,440	18,373
Cost/overall death averted	–	688,431	750,636	733,099	838,180	760,230	−106,231	711,931	497,605	343,025	324,435
Cost/QALY gained	–	35,142	34,166	36,733	33,393	34,856	−4,332	34,999	22,766	15,889	15,275

a Where Q1 month=every 1 month.

b Where negative numbers delineate a cost-savings.

## Discussion

In this regional model of HIV transmission, we found that PrEP implementation in all MSM, irrespective of risk profile, provides important reductions in HIV infections and mortality. Increasing the frequency of HIV testing in susceptible individuals on PrEP resulted in minimal improvement in the number of infections averted, even when testing monthly. When compared to established cost-effectiveness thresholds ($20,000 to $100,000 CAD per QALY) [41], general PrEP delivery to high- and low-risk MSM was not cost-effective.

Focusing PrEP on the highest risk HIV-uninfected MSM was a more efficient strategy than targeting all high and low risk at comparable levels. Specifically targeting a fraction of highest risk MSM (25% coverage) was the most cost-effective option, with a cost per QALY of approximately $35,000 CAD, similar to values previously described in the literature for comparable populations [15,16]. This occurs because even with 25% PrEP coverage, few high-risk MSM (in absolute numbers) remain susceptible to HIV. In contrast, improving PrEP efficacy in high-risk individuals, via better medication adherence [12,13], increased cost-effectiveness. Recent empirical studies suggest the potential for even better efficacy than the 44% efficacy reported in iPrEX [42]. With 99% efficacy, increasing PrEP coverage in high-risk MSM showed even less costs per QALY that could be achieved. Understanding a community's high-risk population (its relative size and relative HIV risk) will be important in guiding endpoints for PrEP implementation programmes, such that coverage targets can be set that optimize cost-effectiveness.

Increasing the frequency of HIV testing in susceptible individuals on PrEP resulted in minimal improvement in the number of infections averted, even when testing monthly. This is because most new infections still occur in non-users when PrEP efficacy is moderate to high. Decreasing the frequency of testing (outside of guideline recommendations of every three months) suggests minimal cost savings and modestly more infections. There has also been documented drug resistance in breakthrough infections [43]. Together, these suggest that it may be more efficient to focus efforts on PrEP adherence rather than testing frequency if there are little marginal benefits.

Given the cost savings of approximately $4,000 CAD per QALY of HIV testing alone in high-risk populations, our findings suggest a potentially large collateral health benefit of PrEP programmes via engaging more high-risk MSM in routine HIV testing, even if they do not ultimately take PrEP. This is likely occurring due to both behavioural changes in those who are diagnosed with HIV and early initiation of ART, both of which will lead to reduced transmission. Some of the barriers to HIV screening among MSM include a belief that they are not at risk for infection, fear of a positive test and fear that other people might find out they are infected [44–47]. Particularly because of its novelty, PrEP programmes offer a new opportunity to engage these individuals in care by providing a means through which they may reduce their likelihood of infection, and potentially feel more comfortable with routine screening. Retention in PrEP programmes, regardless of PrEP initiation, may thus be an important programmatic objective, given the significant cost benefit of having regular screening in high-risk MSM.

Of note, with increased HIV testing in a PrEP programme, the fraction of undiagnosed men with HIV declines, and the number of new HIV diagnoses can outpace incident HIV infections. Since HIV diagnoses are used for surveillance and assessing response to interventions, this could misinterpret the population-effectiveness of PrEP programmes. There may be increased HIV cases diagnosed due to increased HIV testing, especially early in the PrEP programme. Similarly, observed declines in new diagnoses may underestimate true declines in new HIV infections.

### Limitations

While a strength of this study is its regional focus, using a city with epidemic features similar to other urban MSM HIV epidemics [22,48], the findings must be interpreted in this context and are conditional on the assumptions and data inputs. First, we did not include female sexual partners of MSM. Based on available data, the sexual structure of the model did not detail a more nuanced sexual network and behavioural heterogeneity. Second, we tested the PrEP interventions under an endemic equilibrium, which was supported by local stability in the rates of HIV diagnoses and mortality in the last decade [1,4]. It is possible that this equilibrium could be disrupted, and the cost-effectiveness of PrEP may vary during periods of growing or declining epidemics [49]. A third limitation is our assumption that the cost of PrEP medication remains stable, even if adherence is less than daily. Dosing PrEP as infrequently as four times weekly has recently been estimated to still provide nearly 100% risk reduction among MSM [13]. Less frequent dosing and the possibility of future reductions in PrEP drug costs suggest the potential for further cost savings [12]. Fourth, the cost-effectiveness could be overestimated by the low frequency of HIV testing used for this population compared to guidelines. However, the baseline HIV testing rate drew on numbers of performed HIV tests and the susceptible population size [1] and is corroborated by other studies of the same population [35]. Fifth, cost-effectiveness ratios may be over- or underestimated if the quality of life ratio for individuals on PrEP is not equal to one – the value we used in the absence of data to suggest otherwise. Lastly, we assumed ART adherence in those with diagnosed HIV to be excellent and highly effective, which is an increasing reality in the era of well-tolerated once-daily dosing regimens in high-income settings [50]. However, the assumption of perfect ART adherence for those with HIV may overestimate the benefit of a testing-only strategy, and underestimate the potential benefit of PrEP in the susceptible population, whereby individuals with poor ART adherence could actively transmit HIV, increasing the force of infection.

Important next steps include an evaluation of the impact of PrEP use on rates of ART resistance accumulation among those who become infected while taking PrEP, as has preliminarily been explored in some models [51]. Further, given the results of the placebo-controlled IPERGAY trial, in which “on-demand” PrEP (averaging roughly three to four tablets weekly) was associated with an 86% risk reduction [52], future work should assess how best to utilize daily and intermittent PrEP strategies from both a preventive efficacy and cost-effectiveness perspective. Lastly, although clinical data has not suggested significant increases in risk behaviour on PrEP (“risk compensation” or “behavioural disinhibition”) [13,14], the clinical and cost-effectiveness impact of PrEP-related reductions in condom use and increased rates of other STIs warrant further study [53].

## Conclusions

Providing once-daily PrEP for HIV prevention among MSM in a large, urban city could be associated with important reductions in HIV infections and deaths from the use of PrEP itself, and from newly engaging MSM in HIV care through PrEP-related screening. The most efficient approach to PrEP delivery would involve identifying high-risk MSM and aiming for modest PrEP coverage, with a focus on maximizing PrEP adherence over more frequent HIV testing.

## Supplementary Material

Optimizing HIV pre-exposure prophylaxis implementation among men who have sex with men in a large urban centre: a dynamic modelling study
