# Supplementary material for: Optimizing HIV pre-exposure prophylaxis implementation among men who have sex with men in a large urban centre: a dynamic modelling study
Source: J Int AIDS Soc. 2016 Sep 23;19(1):20791. doi: 10.7448/IAS.19.1.20791 (PMC5035769; doi:10.7448/IAS.19.1.20791)
Supplement: Optimizing HIV pre-exposure prophylaxis implementation among men who have sex with men in a large urban centre: a dynamic modelling study [file JIAS-19-20791-s001.pdf]

Supplemental Text

Model description

We developed a compartmental, stochastic-deterministic ('hybrid') mathematical model of dynamic HIV transmission. The model included 24 compartments (*i or j*), representing different states of HIV serostatus, known/unknown diagnosis, activity-level, and ART or PrEP use (Figure 1). Deterministic equations (shown below, equations 1 to 11) were utilized when compartments were >5,000 in size.

If the compartment size fell below 5,000, the compartment was modeled using stochastic approaches. For stochastic processes, deterministic equations were converted to a probability of an event occurring at a single time point. Random numbers were then used to determine if the probabilistic event would occur or not. With stochastic modeling, compartment size increased or declined by discrete integers; however, when switching to deterministic, movement into or out of a compartment occurred using continuous variables. Possible transitions included: staying in a compartment, transitioning to another compartment, being removed from a compartment due to mortality, or entering the susceptible compartment. A compartment size could never be <0.

We calibrated an introduction rate to achieve 1% overall growth in the HIV uninfected population group, in keeping with growth of the Canadian population. We assumed the fraction of MSM who entered the high-activity and the low-activity groups remained constant over time. However, excess HIV-attributable mortality in

the high-activity group (due to higher HIV incidence) did not lead to ‘replacement’ by individuals from the low-activity group.

The model was represented by the following set of coupled, ordinary differential equations (within the deterministic framework). The state variables and symbols are shown in Figure 1, and defined in Tables 1 and S1.

Table S1: Table of Additional Model Parameters

Parameter	Value(s)
Compartment Numbers (i), (j)	1-24
Entry Rate (β_i) Calibrated to Population Growth	1%
Rate of HIV Diagnostic Testing in PrEP (ς)	0.25 (0.083-1)
Population in susceptible compartment (S_i)	-
Population in infected compartment (P_i)	-
Population in PrEP compartment (Pr_i)	-
Population in on-ART compartment (A_i)	-
Total Population (N)	-

Equations

Equation 1. Change in Susceptible population (Compartments 1-2):

$$\frac{dS_i}{dt} = \beta_i - \pi_i S_i \sum_{j=5-14,17-24} \frac{\tau_j \omega_j P_j}{N} - (\delta + \varphi) S_i + \varpi (Pr_{i+2})$$

Equation 2. Change in Uninfected PrEP Population (Compartments 3-4):

$$\frac{dPr_i}{dt} = \varphi S_{i-2} - \pi_i(1-\varepsilon)Pr_i \sum_{j=5-14,17-24} \frac{\tau_j \omega_j P_j}{N} - (\varpi + \delta)P_i$$

Equation 3. Change in Undiagnosed Infected Population (Compartments 5-6):

$$\frac{dP_i}{dt} = \pi_{i-4} S_{i-4} \sum_{j=5-14,17-24} \frac{\tau_j \omega_j P_j}{N} - (\zeta + \eta_i + \delta)P_i$$

Equation 4. Change in Undiagnosed Infected Population (Compartments 7-10):

$$\frac{dP_i}{dt} = \eta_{i-2} P_{i-2} - (\zeta + \eta_i + \delta)P_i$$

Equation 5. Change in Undiagnosed Infected Population (Compartments 11-12):

$$\frac{dP_i}{dt} = \eta_{i-2} P_{i-2} - (\zeta + \delta_a)P_i$$

Equation 6. Change in Infected PrEP Population (Compartments 13-14):

$$\frac{dPr_i}{dt} = \pi(1-\varepsilon)Pr_{i-10} \sum_{j=5-14,17-24} \frac{\tau_j \omega_j P_j}{N} - (\varsigma + \delta)P_i$$

Equation 7. Change in on-ART population (Compartments 15-16):

$$\frac{dA_i}{dt} = \alpha_i \sum_{j=i+2,i+4,i+6,j+8} P_j - (\varrho + \delta)A_i$$

Equation 8. Change in Diagnosed Infected Population (Compartments 17-18):

$$\frac{dP_i}{dt} = \zeta P_{i-12} - (\alpha_i + \eta_i + \delta)P_i$$

Equation 9. Change in Diagnosed Infected Population (Compartments 19-20):

$$\frac{dP_i}{dt} = \zeta P_{i-12} + \varrho P_{i-4} + \eta_{i-2}P_{i-2} - (\alpha_i + \eta_i + \delta)P_i + \varsigma Pr_{i-6}$$

Equation 10. Change in Diagnosed Infected Population (Compartments 21-22):

$$\frac{dP_i}{dt} = \zeta P_{i-12} + \eta_{i-2}P_{i-2} - (\alpha_i + \eta_i + \delta)P_i$$

Equation 11. Change in Diagnosed Infected Population (Compartments 23-24):

$$\frac{dP_i}{dt} = \zeta P_{i-12} + \eta_{i-2}P_{i-2} - (\alpha_i + \delta_a)P_i$$

HIV Transmission

In this model, HIV transmission occurs as a result of sex between men. The likelihood of an individual having a sexual partnership with an individual in a given compartment was proportional to the number of individuals in that compartment. The mixing term is described as follows, where P represents the population of a given compartment, and N the total model population:

$$\sum_{j=5-14,17-24} \frac{\tau_j \omega_j P_j}{N}$$

The activity level (high- or low-activity) of the individuals governed their potential number of sexual partners. Condom use and transmission risk varied by compartment.

The likelihood of HIV transmission between an infected and uninfected male partner, per relationship, per year was defined as τ and could be reduced by condom use ($\boldsymbol{\omega}$) or by PrEP use ($\boldsymbol{\varepsilon}$) (equations 1-3,6). ART use was assumed to reduce transmission to zero. Inherent in this transmission model is the assumption that transmission risk can be stratified into CD4 dependent groupings, which is employed frequently in HIV transmission models. Similarly, we assumed that partnership mixing was proportional to sub-population size. This assumption may be more accurate in a regional model compared to a national model, but still is only an approximation of typically complex interconnected sexual networks, potentially with activity-based assortment. We also assumed that condom use proportionately decreases risk of transmission across all group evenly and consistently. Similarly, we assume that individuals who are diagnosed with HIV have a reduction in 'overall risk' which we denoted as reduction in transmission risk, separate from number of partners.

PrEP and Antiretroviral Treatment

In this model, individuals could move into compartments corresponding to HIV 'prophylaxis' with PrEP (daily tenofovir/emtricitabine), and also compartments corresponding to antiretroviral therapy. Regarding PrEP use, our baseline assumption was a 44% reduction in risk of transmission, which was reflected in the

transmission risk term but not the activity ('number of partners'). We also assessed scenarios of variable PrEP efficacy up to 99%, which may correspond to improved adherence [12]. We did not model the possibility of the introduction of resistance, the magnitude of which in studies has remained small, but could on a population-based scale represent a significant attenuator of effectiveness. In addition, we also assumed that there was no risk compensation with PrEP. Risk compensation has not been observed in organized trials [13]. We also assumed that patients who stop ART were on treatment sufficiently long enough to have recovered a robust CD4 count, and thus they enter into a corresponding compartment of diagnosed/HIV positive. We assumed that patients on treatment with ART have no chance of transmitting virus.

Calibration

This model was calibrated to historical conditions shown in (Table A1, Figure S1), derived from local public health data on HIV infection rate and mortality rate, where population specific values (Toronto MSM) were estimated based on regional rates and historical population proportions. Monte Carlo sampling was employed for calibration, where parameters that were varied (ART on-treatment rates, reduction in risky sexual behavior after diagnosis, mortality in those with AIDS, combined condom use/efficacy, and ART cessation rate) in a random fashion over defined ranges (Table 1). 500 simulations were performed with Monte Carlo sampling, simulating over a time course of 10 years. The parameter set that resulted in stable rates of HIV infection and mortality matching historical ranges for the City of

Toronto over a 10-year period from 2001-2011 [39]. Initial compartment sizes were based on a focused query of data from the Ontario Cohort Study [53], a multi-site provincial cohort of HIV+ individuals, where we evaluated CD4 count at time of ART initiation and extrapolated expected population sizes. The model was then run with population distributions based on equilibrium states and subsequent rates of infection and mortality were confirmed stable over the simulation period and within historical estimates. The model's baseline HIV prevalence ratio of high-risk to low-risk populations was 2.9. Using co-infection as an indicator of high-risk populations, we can compare the prevalence of HIV in the 10% of MSM with syphilis (in a local population), to those without syphilis, and we find a similar prevalence ratio of 3 [35].

When starting the PrEP introduction from equilibrium state, simulations were run out to an additional 20 years, with 150 stochastic runs per parameter set to ensure mean incident diagnosed and undiagnosed HIV infections, varied less than 1% with each additional run.

Figure S1: Estimates of HIV diagnosis rates and HIV deaths by year based on historical data, with model calibrated HIV diagnosis and HIV deaths.

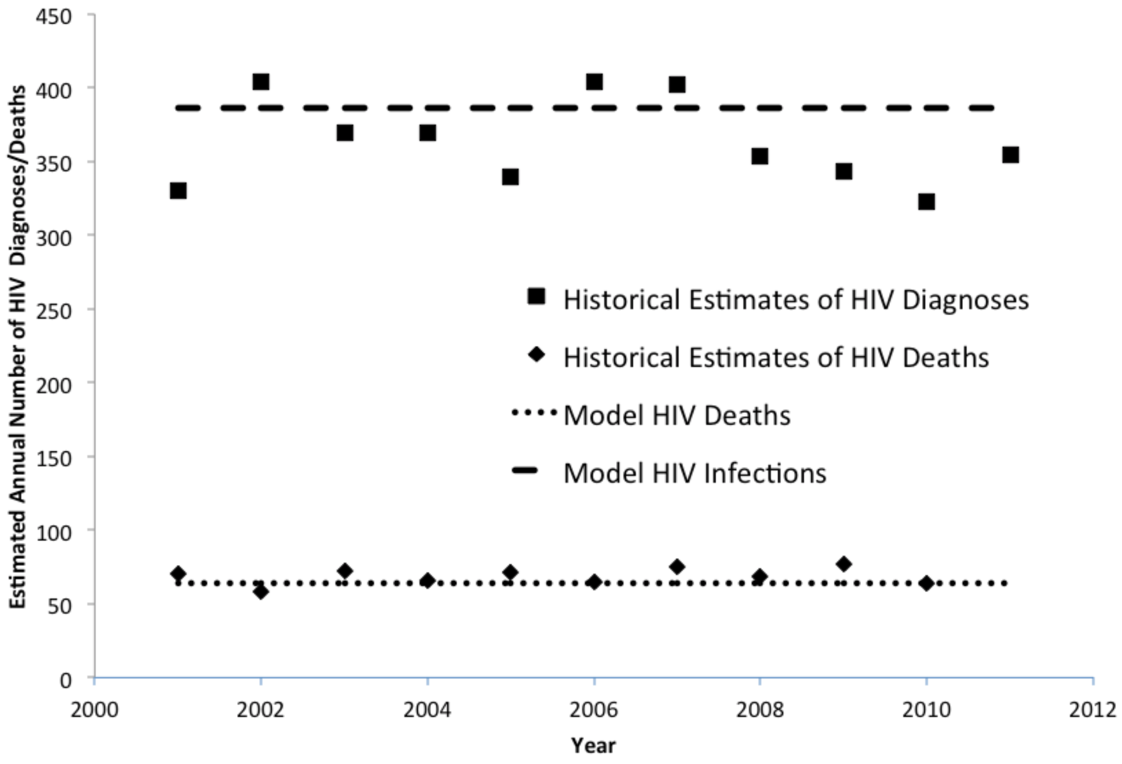

Cost Effectiveness Analysis

We chose to evaluate the cost of PrEP introduction from a health-systems perspective. We used Canadian-based data (Table 1) to estimate the costs associated with HIV-related clinical care (for those on and off treatment), ART and PrEP related drug costs, as well as diagnostic testing costs. We calculated costs at each time step of the model and these were summed and discounted at a rate of 3% per annum. All values were shown in CAD.

In order to determine cost-effectiveness, we also estimated the total quality-adjusted life years (QALYs) gained with different interventions. Using established

quality of life ratios, we determined the total quality adjusted life years, calculated at each time step, and compared years gained with an intervention compared to the baseline scenario. We did not extrapolate our model over longer than 20 years, and thus our estimated incremental quality adjusted life years may be an underestimate of the true value. We also discounted QALYs at a rate of 3% per annum.

Using our health care related costs and QALYs we determined the incremental cost-effectiveness, shown as the ratio of (total incremental costs)/(incremental QALYS) in post-intervention groups compared to baseline groups. These are shown in Figure 2.

Table S2: Initial Conditions and Historical Outcomes [1]

Variable	
Total MSM Susceptible [1]	57,400
Infected Population [1]	10,575
Diagnosed HIV+ [1]	7,598 (71%)
Undiagnosed HIV+ [1]	3,067 (29%)
On Treatment Fraction	63%
Historical Outcomes [1,37]	
Diagnosed Infection Rate	330-400
Mortality Rate	58-72
Observed Outcomes in	

Baseline Model	
Diagnosed Infection Rate	386
Mortality Rate	64

All rates are per annum.
